# Complex lifestyle intervention among inactive older adults with elevated cardiovascular disease risk and obesity: a mixed-method, single-arm feasibility study for RESTART—a randomized controlled trial

**DOI:** 10.1186/s40814-021-00921-0

**Published:** 2021-10-27

**Authors:** Trygve S. Deraas, Laila Hopstock, Andre Henriksen, Bente Morseth, Anne Sofie Sand, Inger Njølstad, Sigurd Pedersen, Edvard Sagelv, Jonas Johansson, Sameline Grimsgaard

**Affiliations:** 1 Department of Community Medicine, UiT The Arctic University of Norway, Tromsø, Norway; 2 School of Sport Sciences, UiT The Arctic University of Norway, Tromsø, Norway; 3 Department of Health and Care Sciences, UiT The Arctic University of Norway, Tromsø, Norway

**Keywords:** Feasibility study, Older adults, Lifestyle intervention, Primary prevention, Cardiovascular disease risk, Obesity, Exercise therapy/methods, Resistance training, Diet, Behavioural counselling

## Abstract

**Background:**

Physical inactivity and obesity are global public health challenges. Older adults are important to target for prevention and management of disease and chronic conditions. However, many individuals struggle with maintaining increased physical activity (PA) and improved diet. This feasibility study provides the foundation for the RESTART trial, a randomized controlled trial (RCT) to test a complex intervention to facilitate favourable lifestyle changes older adults can sustain. The primary objective of this study was to investigate study feasibility (recruitment, adherence, side-effects, and logistics) using an interdisciplinary approach.

**Methods:**

This 1-year prospective mixed-method single-arm feasibility study was conducted in Tromsø, Norway, from September 2017. We invited by mail randomly selected participants from the seventh survey of the Tromsø Study (2015–2016) aged 55–75 years with sedentary lifestyle, obesity, and elevated cardiovascular risk. Participants attended a 6-month complex lifestyle intervention program, comprising instructor-led high-intensive exercise and nutritionist- and psychologist-led counselling, followed by a 6-month follow-up. All participants used a Polar activity tracker for daily activity monitoring during the intervention. Participants were interviewed three times throughout the study. Primary outcome was study feasibility measures.

**Results:**

We invited potential participants (*n*=75) by mail of which 27 % (*n*=20) agreed to participate. Telephone screening excluded four participants, and altogether 16 participants completed baseline screening. The intervention and test procedures of primary and secondary outcomes were feasible and acceptable for the participants. There were no exercise-induced injuries, indicating that the intervention program is safe. Participants experienced that the dietary and psychological counselling were delivered too early in the intervention and in too close proximity to the start of the exercise program. Minor logistic improvements were implemented throughout the intervention period.

**Conclusion:**

This study indicates that it is feasible to conduct a full-scale RCT of a multi-component randomized intervention trial, based on the model of the present study. No dropouts due to exercise-induced injury indicates that the exercises were safe. While minor improvements in logistics were implemented during the intervention, we will improve recruitment and adherence strategies, rearrange schedule of intervention contents (exercise, diet, and psychology), as well as improve the content of the dietary and behavioural counselling to maximize outcome effects in the RESTART protocol.

**Trial registration:**

ClinicalTrials.gov Identifier: NCT03807323 Registered 16 January 2019 – retrospectively registered.

## Key messages

### What areas of uncertainty regarding feasibility existed prior to this study?

Several uncertainties needed clarification before proceeding to a costly, full-scale multi-component RCT.

We examined the recruitment process, participant attendance, and adherence to study activities, and whether participants experienced adverse events and exercise-induced injuries. We evaluated tools for screening of participants, data collection, and measurement of physical activity, as well as study logistics and study team performance.

### What are the key findings on feasibility from this study?

The recruitment response fulfilled the pre-specified criteria, but there is room for improvement. Participant attendance and adherence was fair, and there were no dropouts due to exercise-induced injuries.

Participants’ interviews showed that the social interaction was important and that the timeline and content of the various intervention components needed revision. Tools for data collection performed well. We also identified need for minor adjustments of study logistics and study team performance.

### What are the implications of the findings on the design of the main study?

We found that it is feasible to conduct a full-scale RCT of a multi-component intervention, with some adaptations on the feasibility study model. We will extend the recruitment approach and adjust the schedule and content of intervention components. Finally, we will adjust study logistics and study team organization.

## Background

Physical inactivity is a well-documented independent risk factor for cardiovascular disease (CVD) and type 2 diabetes [1], as well as CVD risk factors such as obesity [2], hypertension [3], and atherosclerosis [4]. Physical inactivity increases with age, and a minority of the population adheres to the current physical activity (PA) guidelines [5, 6]. The world faces an obesity epidemic increasing the morbidity and mortality of non-communicable diseases. Thus, increasing PA and reducing obesity are important global public health goals [5, 7, 8]. Older adults represent an important target group for PA interventions to prevent and manage chronic conditions and diseases. Physical activity with focus on endurance, strength, and mobility improves muscle, cardiorespiratory, and metabolic health and reduces risk of functional decline in older adults [9, 10]. Combined weight management randomized controlled trials (RCT) that include both PA and dietary intervention programs are more efficient for weight loss in overweight adults, compared to diet or exercise interventions alone [11].

Exercise levels tend to decrease after the end of an intervention, and participants with obesity and high CVD risk frequently return to baseline weight and PA levels after intervention cessation [12–14]. Successful interventions to promote long-term change have used specific strategies to promote sustainable effects of interventions [15]. An intervention aiming at sustainable lifestyle change must therefore include components that facilitate maintenance of PA levels and dietary changes over time. A meta-analysis of studies involving adults aged above 65 years showed that interventions that include behavioural or cognitive components increase PA levels [16].

RCTs that examine complex interventions for sustainable lifestyle change are scarce. In light of this, our long-term aim is to develop and test a complex intervention, including (1) endurance and strength training, (2) dietary counselling, and (3) habit change counselling to facilitate lasting lifestyle changes in a randomized controlled trial (RCT).

The main purpose for the planned RESTART (*Re*-inventing *St*rategies for healthy *A*geing; *R*ecommendations and *T*ools) trial is to examine whether participants with obesity and physical inactivity can achieve a persistent lifestyle change that increases their PA and improve their quality of life and CVD risk in the long run.

However, to conduct an RCT with several interventions is complicated. We therefore conducted a feasibility study. The primary study aim was to examine whether the intervention is feasible to progress to a full-scale RCT with regard to (1) recruitment, (2) adherence, and (3) side-effects. The secondary aims were to evaluate tools for data collection and measurement of PA as well as screening, study logistics, and team cooperation.

This paper outlines the aims, design, and feasibility study experiences, whereas change in obesity, physical activity, diet, cardiorespiratory fitness (expressed by V̇O_2max_), cardiovascular risk, quality of life, self-efficacy, and self-regulation capacity is described elsewhere [17].

## Methods

### Study design

This feasibility study is reported according to the CONSORT guidelines for pilot and feasibility trials [18]. We conducted a complex 1-year mixed-method, prospective single-arm feasibility study in Tromsø, Norway, from September 2017. The study consisted of a 6-month intervention program (October 2017–March 2018) comprising instructor-led high-intensive exercise and dietary and habit change counselling, followed by a 6-month follow-up with daily activity monitoring by a Polar activity tracker (AT) (March 2018 to September 2018). Each participant was interviewed three times through the intervention and follow-up period.

### Feasibility

Evaluation criteria used for deciding whether to proceed to a full-scale RCT were thematically organized as: (1) recruitment and enrolment and (2) adherence and side-effects. Based on previous experience, we expected 25 % positive response to postal invitations, and that few responders would be excluded during telephone and baseline screening. We outlined several success criteria for these aspects (Table 1).

**Table 1:** Pre-specified criteria for recruitment

Evaluation criteria	Goal	*N*
Recruitment process (*n*=75)		
Postal invitation positive response	> 0.25	19
Eligible after telephone screening	> 0.20	16
Eligible after baseline screening	> 0.18	13

We expected 75 % overall group mean attendance to the group exercise sessions and higher mean group attendance to the three dietary and habit change counselling group sessions, respectively [19]. We further considered a dropout rate up to 20% to be acceptable and expected that less than 10% would drop out due to exercise-induced injury (Table 2).

**Table 2:** Pre-specified criteria for adherence and side-effects

Intervention adherence and side-effects among	Goal
Mean attendance to exercise sessions	> 0.75
Mean attendance to three dietary counselling sessions	> 0.80
Mean attendance to three habit change counselling sessions	> 0.80
Attrition from study	< 0.20
Dropouts due to exercise-induced injury	< 0.10

We monitored screening and study logistics, study team cooperation, and data collection, to improve the setup of a future RCT. Finally, we interviewed participants at three time points to obtain their feedback on study participation.

### Recruitment, screening, and enrolment of participants

To investigate study feasibility, we needed to enrol a minimum of 12 participants, similar to the actual size of an exercise intervention sub-group in a full-scale RCT.

Participants were recruited from the Tromsø Study at UiT The Arctic University of Norway, an ongoing prospective population-based study in the municipality of Tromsø, Norway. Altogether, seven surveys were conducted from 1974 to 2016, with extensive data collection, including questionnaires, interviews, measurements, clinical examinations, and biological samples. We invited randomly selected participants from the seventh survey of the Tromsø Study 2015–2016 (Tromsø 7) using the following criteria: (1) age 55–75 years; (2) self-reported sedentary leisure time PA, as defined by the Saltin–Grimby Physical Activity Level Scale (inactive: reading, TV watching, movies, or doing other sedentary activities during leisure time) [20]; (3) obesity (body mass index (BMI) ≥30 kg/m^2^); (4) no self-reported previous myocardial infarction; and (5) medium or high 10-year risk of first-time cardiovascular disease event calculated by NORRISK 2, a Norwegian adaptation of the SCORE CVD risk model, validated using Norwegian population samples [21]. An invitation including study information was sent by mail. A trained research nurse screened all responders via telephone according to criteria listed in Table 3.

**Table 3:** Telephone screening inclusion criteria

Question	Inclusion criteria
Have you had a heart attack or hospitalization of any other disease since you participated in Tromsø 7?	No
Do you have any known diseases making activity challenging or impossible to carry out?	No
Is there any reason that you shouldn't follow an activity program even if you wanted to?	No
Do you have any physical problems with getting up out of bed or chair?	No
Do you have any physical problems with squatting?	No
Can you put your hands over your head or shoulder height?	Yes
Would training with 20 others in a group at a fitness centre during a quiet period of the day be ok to you?	Yes
Do you have the opportunity to attend for group sessions between 1415–16 and 1445–1615 Tuesdays/Thursdays?	Yes
Can you meet up to six group sessions during the first ten weeks?	Yes
The project requires you to wear training clothes on group activities; it is okay?	Yes
Do you have a smartphone android or iPhone?	Yes
Is it ok for you to use a Polar activity tracker most days/hours during the project period	Yes

Telephone-screened participants received an invitation letter including study information and several comprehensive questionnaires. A written informed consent to participate in the study was obtained when they met for further screening sequentially at three sites: The Clinical Trial Unit (CTU) at the University Hospital of Northern Norway (UNN), the UiT exercise research laboratory at Alfheim stadium, and Stamina health clinic downtown Tromsø. Table 4 outlines the eligibility criteria at baseline screening.

**Table 4:** Eligibility criteria at baseline screening

Inclusion criteria	
Age 55–75 years	
BMI ≥ 30 kg/m^2^	
Medium to high NORRISK 2 score^a^	
Motivation for lifestyle change	
Has consented to participate and agreed not to participate in another study during the study period	
Exclusion criteria	
Self-reported	
Active disease with short life expectancy	
Diseases that limit project participation/physical activity including serious mental or cognitive impairment	
Previous myocardial infarction	
EKG showing severe rhythm disturbance, unstable angina or heart attack	
Blood pressure or blood tests result indicating serious disease	

^a^Ten-year risk for acute heart attack or stroke, including cardiovascular death (medium to high risk): > 8 % risk for persons at age <65 years, >12 % risk for persons at age 65–74 years

### Measurements

The study included four visits: Baseline screening (BS), 3 months after start of intervention (V1), 6 months after start of intervention (V2), and 6 months after end of intervention (V3). Several structured questionnaires, clinical and instrument-based examinations, blood tests, urine samples, and semi-structured interviews were scheduled at different visits as shown in Table 5.

**Table 5:** Study activity and data collection schedule

Activity/year	Invitations and phone interviews	Baseline screening and consent	Start exercise	2017											2018				
Week in study	1–3	3–4	5	6	7	8	9	10	11	12	13	14	15	16–17	18–19	20–27	28–29	36, 43, 50	56–57
Measurement point		BS													V1		V2		V3
Questionnaires		x													x		x		
Food frequency questionnaire (FFQ)		x															x		
Diet (3-day registration)		x																	
Measurements, clinical examinations, blood tests, physical function tests		x													x		x		x
Exercise			x	x	x	x	x	x	x	x	x	x	x	x	x	x			
Individual nutrition counselling			x	x															
Nutrition group session						x				x			x						
Habit change group session							x		x			x							
Activity monitor manual download				x			x			x			x		x		x	x	x
Semi-structured interviews															x				x
Focus group interviews																	x		

### Questionnaire data

Information on diet was recorded at BS and V2 by a validated food frequency questionnaire (FFQ) described elsewhere [22]. Data were imported into the food and nutrient composition database and calculation system KBS (KBS, version 7.3, database AE14, University of Oslo, Oslo, Norway) where energy, nutrients, and food groups were calculated.

We used an in-house questionnaire to obtain information on education, social network, chronic diseases, symptoms and ailments, diseases in the family, use of medication, smoking status, and alcohol consumption. The questionnaire also included full versions or specific items from validated instruments to measure PA by the “Saltin–Grimby Physical Activity Level Scale” [20, 23], the self-reported physical activity questionnaire in the Nord-Trøndelag Health Study (HUNT 1) [24] and the International Physical Activity Questionnaire (IPAQ) [25], sleep by the Bergen Insomnia Scale [26], satisfaction with life by the Satisfaction With Life Scale [27], self-esteem by the Rosenberg Self-Esteem Scale [28, 29], self-efficacy by the General Self-efficacy Scale (GSE) [30, 31], mental health by the SCL-10 [32, 33], and self-reported health by EQ-5D-5L [34].

### Physical capacity testing

All measurements were performed using standard methods by trained test personnel.

#### Cardiovascular and lung function

Blood pressure, resting heart rate, resting electrocardiogram (ECG), and lung capacity were measured at time points BS, V1, and V2. Blood pressure and resting heart rate (HR) were measured on the right arm of all participants by a Dinamap ProCare 300 monitor (GE Healthcare, Norway). The mean of the last two blood pressure measurements and the last HR measurement were used as measured values. Measurements were recorded three times at 1-min intervals, after a 2-min seated rest. Spirometry was performed using Diagnostica Spirare spirometry model SPS 330 (Diagnostica AS, Norway).

#### Cardiorespiratory fitness

Maximal oxygen uptake (V̇O_2max_) was measured at time point BS, V1, and V2 during walking/running on a treadmill. An incremental test to exhaustion was performed to assess V̇O_2max_ on a motorized treadmill (Woodway GmbH, Weil am Rhein, Germany). To safeguard participants, ECG recordings were conducted at CTU prior to the test visit. Further, the test personnel asked participants two questions before start of the test: (1) “Has your doctor ever told you that you have heart disease that impedes intense physical activity?” and (2) “Have you lately been breathless, or have you experienced chest pain when walking uphill?”

During the test, the participants wore a Polar RS400 heart rate (HR) monitor (Polar Oy, Finland) connected to Polar H10 HR sensor chest belt (Polar Oy, Finland), and a face mask connected to Cosmed K5, a portable ergo spirometry system (Cosmed SRL, Rome, Italy) set in mixing chamber mode, which was placed on the participants’ back. The test personnel regularly encouraged the participants to continue to exhaustion.

The starting speed and incline (0–4%) for the incremental test to exhaustion were based on a subjective assessment by the test personnel, by evaluating the respiratory exchange ratio (RER) during the warmup. Thereafter, the speed was set to either 4 or 5 km·h^−1^, and the inclination of the treadmill increased with 2% every second minute until exhaustion. Thirty seconds before the next increase in incline, the participants answered if they could manage an increase of incline with 2% with a thumb up or down indicating yes or no, respectively. When refusing an increased incline, the participants were instructed to keep walking until they felt exhausted and signal that they wanted to stop. Ventilatory parameters and pulmonary gas exchange were measured every 10 s, and a RER ≥ 1.05 was, in combination with a plateau of the V̇O2 –work rate curve, used to determine if the participants reached V̇O_2max_ [35]. V̇O_2max_ was calculated as a mean of the three highest consecutive 10 s measurements.

#### Strength testing

We tested participant’s maximum dynamic muscle strength in three exercises at BS and V2, according to the protocol for testing one repetition maximum (1 RM) [36], which was defined as the heaviest weight the participant could handle: (1) Squat (IT7006 45° Leg Press/Hack Squat), (2) seated pulldown (Technogym selection pro LAT Vertical Traction), and (3) seated incline chest press (Technogym Pure Strength - Incline chest press).

We tested the participant’s rate of force development (RFD) at BS and V2 by mounting a pressure sensor plate (ALU4 2003, HUR Labs Oy, Kokkola, Finland) on the footplate of the squat testing equipment. The sensor plate was connected to a portable laptop through a USB cable and monitored with the manufacturer’s software (Force platform software suite, HUR Labs Oy, Kokkola, Finland).

#### Flexibility and balance

Maximal passive range of motion in neck, shoulder, hip, knee, and ankle were measured at time point BS and V2. The range of motion was tested by goniometry. Testing protocol was developed according to Norkin and White [37]. Balance was planned to be measured at BS and V2 by “one-foot standing” test as maximal time standing on one foot with closed eyes [38] as a part of the flexibility test session.

### Blood parameters

Non-fasting venous blood samples were collected with standard methods and consecutively analyzed at the Department of Laboratory Medicine, UNN. Haemoglobin, glucose, glycated haemoglobin (HbA1c), total cholesterol, high-density and low-density lipoprotein (HDL and LDL) cholesterol, triglycerides, creatinine, creatine kinase (CK), alanine aminotransferase (ALAT), aspartate aminotransferase (ASAT), gamma glutamyl transferase (GGT), and vitamins D2, D3, and 25-hydroxy vitamin D were analyzed at BS, V1, and V2. Thyroxin (FT4) and thyroid stimulating hormone (TSH) were analyzed at BS.

### Adiposity

Adiposity was defined by anthropometric measures: BMI (body weight in kilograms divided by height in meters squared (kg/m^2^)), waist circumference (measured at the umbilical level in centimetres by a measurement tape), and body composition (body fat and lean mass) measured by dual-energy X-ray absorptiometry (DEXA) (Lunar GE Prodigy Advance, GE Medical Systems).

### Activity and heart rate measurements

#### Activity tracker

One week prior to intervention start, all participants received a Polar M430 AT (Polar, Kempele, Finland) to collect PA and HR data. A Polar user account was created for each participant and included information from baseline screening on BMI and maximum HR. Privacy was maintained by not disclosing the login credentials to participants, only storing non-identifiable information on the account, and by deactivating the AT GPS (global positioning system). Participants were instructed to wear the AT all day and all night for 6 months, and only take it off during re-charging on Sundays. Participants wore a heart rate sensor chest belt (Polar H10) during the exercise sessions to obtain accurate HR measurements. HR zones followed Polar standards: Very Light (51–60% of HR_max_), Light (61–70% of HR_max_), Moderate (71–80% HR_max_), Hard (81–90 % of HR_max_), and Very Hard (91–100% of HR_max_). The chest HR sensors used at exercise sessions were stored at the gym and handed out to the participants before each session. At the end of intervention, all participants were invited to wear the watch and continue to share data for six additional months.

In addition, participants wore the AT without heart rate sensor in daily free living. The Polar device provides daily estimates for minutes of sleep, sedentary time, light physical activity, moderate physical activity, vigorous physical activity, and non-wear. We calculated wear time by adding minutes of sedentary time and light, moderate, and vigorous physical activity. Each participant contributed daily information on step count, energy expenditure, wear time and number of minutes in sleep, sedentary behaviour, and light-, medium-, and vigorous PA, respectively. Only data from “valid days,” defined as at least 10 h of wear time according to Troiano et al. [39], were included in analysis.

#### Accelerometer

An ActiGraph wGT3X-BT (ActiGraph, LLC, Pensacola, USA) accelerometer was worn at the right hip to measure PA and sedentary behaviour for 24 h for eight consecutive days at BS and V2. The participants were instructed to perform their usual daily activities and only remove the accelerometer when performing water activities. We chose a 24-h protocol to align our data collection with the last waves of the Tromsø Study, from where we recruited current study participants and where we will recruit our sample for the upcoming full-scale RCT [6].

ActiLife software (ActiGraph, LLC, Pensacola, USA) version 6.13.3 was used for device initialization, setup (100 Hz sampling rate), and data download (10-s epoch, 3-axes). Ten-second epochs were used as this was the lowest possible setting. Epochs were further aggregated to 60-s epochs before applying the VM cut-offs [6]. The ActiGraph was setup with 100 Hz sampling rate, the highest possible frequency. This is recommended by Migueles et al. [40] to enable future research to retrospectively use or improve data analysis. We used normal frequency filtering. We chose a minimum of 4 valid days and 10 h per day, as this is established as a standard in most epidemiological studies using accelerometer [41].

Non-wear time was identified using Hecht et al. [42] wear time algorithm and excluded from analysis. We calculated intensity variables using triaxial activity count (i.e., vector magnitude (VM)) cut-off suggested by Sasaki et al. [43], Kozey-Keadle et al. [44], and Peterson et al. [45]. Each minute of activity was classified as sedentary behaviour (≤ 149 VM), light PA (150–2689 VM), moderate PA (2690–6166 VM), or vigorous PA (≥ 6167 VM). Moderate and vigorous PA was further combined into one variable for moderate-to-vigorous PA.

### Interviews

The main focus of the study was to investigate how participants experienced the various intervention measures and to reveal potential improvement when planning an RCT. Individual semi-structured interviews were conducted by a trained researcher at V1 and V3. By using a lifeworld perspective as described by Kvale and Brinkmann [46], the participants’ experiences and points of view were illuminated [47, 48].

We developed an interview guide with fixed predefined questions to ensure that key topics/questions were covered in the interviews. The questions were not necessarily given in the same order in all interviews, and it was possible to record and/or follow up on other relevant topics that arose during the interviews. Main topics during the interviews were attitudes and subjective norms towards health and activity before study start, motivation and barriers to exercise or to be more active, motivation and barriers to have a healthier diet, study participation experiences, lifestyle changing factors, effect of intervention on further lifestyle change, and experiences with the intervention.

At V2, the participants gathered for two focus group interviews. The main topics of these focus groups were similar as in the interviews at V1. All interviews were audio recorded.

### Data storage

The CTU at UNN used REDCap electronic data capture tools for study data collection and management. REDCap (Research Electronic Data Capture) is a secure, web-based application designed to support data capture for research studies, providing (1) an intuitive interface for validated data entry, (2) audit trails for tracking data manipulation and export procedures, (3) automated export procedures for seamless data downloads to common statistical packages, and (4) procedures for importing data from external sources [49].

Secured data were transferred to the Department of Community Medicine, UiT while questionnaire data were manually entered into the same secured database.

### Compensation to the participants

The participants received a gift card (value 40€) to compensate for travel expenses related to participation at V2.

### The intervention

The intervention comprised altogether 22 weeks starting from week 5 of the study. During the first 10 intervention weeks, nutritional education sessions based on the Nordic Nutrient Recommendations [50], and psychologist-led group counselling sessions based on “Implementation Intentions” theory [51] were carried out in addition to group-based exercise.

The participants themselves chose the appropriate time of attendance at the various group sessions. During the last 12 weeks, participants engaged in exercise sessions only.

More detailed description of the intervention program:

*Exercise* focused on endurance, strength, balance, and flexibility, and included two instructor-led sessions per week at the private enterprise “Stamina Trening,” Tromsø, see Table 6 for exercise intervention plan details.

**Table 6:** Exercise intervention plan

Exercise type (weekday)	Week no. (stage)	Total session duration (minutes)	Exercise intensity (% of max HR/% of 1RM^a^) + interval length	Focus
Bike spinning (Thursdays)	1–3 (1)	<35	<80%	Introduction to exercise theory, familiarize with exercise program
	4–5 (2)	<42	Slightly increased	Preparing for high intensity
	6–11 (3)	<50	85%/<3 min	Increase stamina
	12–18 (4)	<55	85–92%/<4 min (4×4 intervals)	Increase stamina
Brisk walking uphill (Thursdays)	19–22	<60	85–92%/<4 min (4×4 intervals)	Adaption to outdoor activity, increase stamina
Aerobic hall sessions (Tuesdays)	1–3 (1)	Aerobic: 20–25 Strength: 20–25 Total: 40–50	<80%	Introduction to exercise theory, familiarize with exercise program
	4–5 (2)	Aerobic: 20–25 Strength: 20–25 Total: 40–50	Slightly increased	Rhythmic aerobic exercises on step box 45–50s work/15–10s break; 3–4 exercises on each leg 3–4 times. Exercise beat 120–130 bpm^b^
	6–11 (3)	Aerobic: 20–25 Strength: 25–30 Total: 50	85%/<3 m Pulse step Borg scale 14–15	Rhythmic aerobic exercises on step box 45–50s work / 15–10s break; 3–4 exercises on each leg x 3–4 times. Exercise beat 120–130 bpm. When increased stamina, heighten step/increase weights
	12–22 (4)	<50	85–92%/<4 min	Short intervals ad modum Tabata (20s intensity/10s pause) 4x4 + strength on the lower body
Maximum resistance training exercise	1–5 (1)	10	80% of 1 RM	Instructor-led hack-squat from extended knee to 90 degrees knee arch. 3x5 repetitions, start at 85% of 1RM. When able to perform > 5reps; increase by 2.5–5 kg. Approx. 3-min rest. Instructed to perform concentric phase fast, eccentric phase slow
	6–22 (2)	10	80% of 1 RM	Peer-supervised, episodic instructor supervised
Daily activity	1–22			Encouraging increase at every instructor-led session

^a^One repetition maximum

^b^Beats per minute

Each Tuesday, participants did aerobic hall sessions indoors with various bodyweight or light dumbbells exercises, emphasizing endurance, balance, flexibility, and muscular strength. Each Thursday, the participants attended indoor bicycle spinning sessions mainly to increase endurance. The indoor spinning session protocol involved 19 supervised and progressively challenging sessions based on a high-intensity interval training (HIIT) model, is obviously time efficient, and has shown superior to moderate intensity exercise for increasing aerobic capacity in healthy older adults [52] and in patients with coronary artery disease [53].

During the last 2 weeks, spinning was replaced by outdoor exercise, preparing the participants for brisk walking based on the 4×4 interval model [54]. Finally, during group exercise sessions, all participants carried out 10 min of resistance exercise to improve maximum strength in the leg and hip extensor muscles in the hack-squat apparatus also used for testing. Half of the participants carried out the exercise *before* the group training started and half of the group *after* the workouts.

During group exercise sessions, the instructors would regularly encourage the participants to increase and to be involved in various daily activities. Each participant’s attendance to different sessions were recorded, as the effects of endurance HIIT may partially depend on how well participants follow the intended exercise protocol. We evaluated protocol adherence during multiple cycling-based interval exercise sessions where participants received “live” instructions using a Technogym projector system. Data on protocol adherence were extracted from 7 sessions evenly distributed throughout the study period.

The two exercise instructors systematically recorded any adverse events and reasons for participant-notified non-attendance. They also advised the actual participant to consult their general practitioner (in a patient list system) for further examination and /or treatment for any health complaints. The project leader received continuous updates from the exercise instructors.

*Dietary intervention* was based on Norwegian dietary recommendations published by the Norwegian Directorate of Health and based on the Nordic Nutrition Recommendations 2012 [50]. The main objective of the nutrition intervention was to enable participants to achieve long-term reduced energy intake and healthier eating habits. All participants completed a 3-day food diary before one individual counselling session with a nutritionist during the first 2 weeks of the intervention. After the individual session, the nutritionist met with two groups of eight participants each during three sessions, weeks 8, 12, and 15 in study. The first focused on general food knowledge, the second on theories for practical cooking, and the third on advice for food purchasing.

*Habit change intervention* included three group sessions of eight participants each led by a psychologist, in weeks 9, 11, and 14 in study. The psychologist introduced the participants to mental exercises according to “Implementation-intention strategies” [55] to increase their awareness of automated actions and to create new situational actions in a more desirable way linked to their lifestyle goals use of to change behaviour, so called “action plans.” When properly formulated and memorized, “action plans” are shown to affect participants behaviour by creating new links between specific situations and actions. Participants were encouraged to develop, test, and discuss one individual action plan related to PA, and one related to diet change by linking a critical situation to an action that they wanted to perform in this situation (e.g., “Whenever my grandchildren visit, I will suggest a walk to the close-by playground”) [56]. The purpose was to prepare participants to behave in line with their new plans when they face these situations in everyday life.

### User involvement and steering group

Patient and public involvement (PPI) in health research is important to enhance the quality and relevance of research [57]. The project steering group included two user representatives. The representative from The National Association for Heart and Lung Disease (LHL) contributed with patient experience as well as practical advice on study planning and conduct. Second, a representative of the municipality of Tromsø also contributed experiences from a complex lifestyle intervention aimed to reduce sick-leave from work.

### Statistical analysis

Feasibility including recruitment, participants’ adherence, and side-effects were investigated by comparison with the pre-specified feasibility criteria (Tables 1 and 2). Feasibility criteria for various primary outcomes are reported.

Recruitment aspects and exercise-induced injury dropout rate were calculated by Wilson score interval to estimate 95% confidence intervals (CI) for proportions [58].

Feasibility criteria for various secondary outcomes are also reported. AT wear time is reported as percentage of participants with high use of AT. Attendance to various intervention activities and interviews is reported as mean session attendance with 95% CI. For nominal data, raw count (number, %) are reported. Deviations from the cycling-based exercise protocol using HR monitors were analyzed with coefficients of variance (CVs), where a higher percentage indicates a larger deviation from the intended HR-based exercise plan. Additionally, paired *t* tests were used to evaluate changes in protocol adherence between the first and last session. A *p* value of <0.05 was considered statistically significant.

A researcher trained in qualitative research methodology performed verbatim transcriptions of the interviews except for the interviews at V3. A professional enterprise, DigForsk AS Kirkenes, performed the verbatim transcription from these interviews. All transcriptions were performed within weeks from the interviews. We used the computer software QSR NVivo 12 Plus (QSR International, Pty Ltd) as a tool for structuring data in the analysis process.

## Results

### Recruitment of participants

#### Postal invitations and telephone screening

We sent an invitation letter to 75 potential participants by mail on 6th of September 2017, to which 20 (27%) agreed to participate. No reminders were sent. Respondents received a detailed description of the project, including risks and potential benefits of participating, underlining that they could withdraw from the study at any time without justification. Participants signed an informed consent before baseline screening. They received a gift card (value 40€) to compensate for travel expenses related to participation. Two respondents were excluded in the telephone interview because they were unable to attend daytime exercise sessions, and two responders were excluded due to functional level.

Thus, we included sixteen out of 75 invited to meet for extended inclusion screening, indicating that 21% of the invited passed telephone screening.

#### Enrolment and baseline screening

**Fig. 1:**
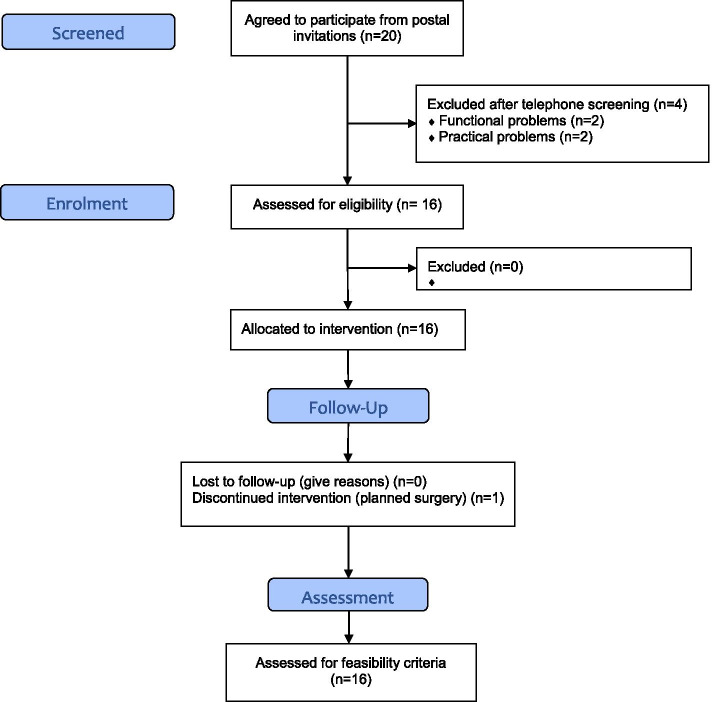
Recruitment and retention information flowchart

From September 25, 2017, all 16 respondents (5 women and 11 men, aged 57–74 years) that passed telephone screening attended baseline screening at the CTU, UNN. All were found eligible (21% of those invited). During functional testing by two certified physiotherapists/personal trainers, two participants were judged to be too restricted in movements to fulfil the inclusion criteria. The two respondents were however highly motivated and were therefore allowed to join the study. They participated in bike spinning exercise, strength training, and dietary and habit change counselling and exercised on their own on days with aerobic hall training. Recruitment and retention information is displayed in Fig. 1 while Table 7 shows key baseline characteristics of the participants.

**Table 7:** Participant characteristics at baseline

Characteristics	Value
Age, years	64.5 (57–74)
Male sex, %	68.8 (11)
Cohabitant, %	62.5 (10)
Education	
University ≥4 years, %	37.5 (6)
University <4 years, %	25.0 (4)
Upper secondary, %	25.0 (4)
Primary, %	12.5 (2)
Daily smoker^a^	
Current, %	20.0 (3)
Previous, %	53.3 (8)
Never, %	26.7 (3)
Hypertension, %	18.7 (3)
Hypercholesterolemia, %	50.0 (8)
Anxiety/depression^b^, %	14.3 (2)
Self-reported health^b^	
Very good, %	7.1 (1)
Good, %	64.3 (9)
Neither good nor bad, %	21.4 (3)
Bad, %	7.1 (1)

Values are median (range) or percentage (number). Hypertension: blood pressure ≥ 140/90 mmHg; Hypercholesterolemia: total serum cholesterol ≥ 5.0 mmol/L; Anxiety/depression: Hopkins Symptom Checklist ≥1.85. ^a^Missing values: 1 participant. ^b^Missing values: 2 participants

### Attendance

#### Return of questionnaires and screening and follow-up tests

Most participants returned questionnaires (14 at BS and V1, and 15 at V2) and 3-day food diary (16 at BS, and 15 at V2). All participants met at the CTU-screening at BS, V1, and V2, while 14 met for treadmill test at BS and V2. All met for strength test at BS and 14 met at V2.

#### Group exercises

Mean attendance rate at exercise group sessions was 0.70 (95% CI (0.60, 0.80)) for all 16 participants. Participants preannounced their absence in 71% of the cases. One of two participants with reduced mobility had one of the highest attendance rates, while the other met for 51% of the exercises. Altogether, 75% attended more than 65% of sessions. The attendance in outdoor activity was low, partly due to slippery and icy roads that required outdoor shoes with spikes.

#### Dietary and habit change counselling

All 16 participants attended one individual dietary counselling session, while 12 participants (75%) attended each dietary group session. Fourteen participants attended two or three dietary group sessions, whereas six participants attended all sessions.

At the habit change counselling group sessions, mean attendance was 79%, 13 participants attended two or three sessions, and nine participants attended at all habit change group sessions. Fifteen participants met at the last habit change group session.

### Interviews

All participants attended interviews at V1 and V3, while 14 out of 16 met for focus group interviews after V2.

### Side-effects and adverse events

No participants dropped out of the intervention program due to exercise-induced injury. Four participants with prevalent musculoskeletal conditions were given alternative exercises due to a short-term worsening of pain. One participant dropped out after 18 weeks due to planned lower limb surgery. One participant was diagnosed with chronic obstructive pulmonary disease (COPD) at a lung specialist clinic after baseline spirometry screening. Two participants reported episodes of cold and seasonal flu during the intervention, resulting in lower participation in exercise sessions during the winter months.

### Process evaluation of logistics

The cooperation between the study nurse at the CTU, the project administrator, and the project leader generally worked well. Study logistics and implementation were demanding as the study team comprised altogether 14 individuals with various tasks at three different test sites. The project leader systematically collected and processed input and experiences from team members and study team meetings. This information was used to improve logistics and cooperation throughout the entire study period. Time scheduled at each test station was too tight, as some participants needed breaks between tests. Responsibility for notifying participants of the time and place of upcoming tests was not clearly stated. Thus, a time schedule card for each participant was introduced at V1. The map guide and marking of entrance doors at different test stations were also improved for V1 and V2.

Finalizing study questionnaires was more time consuming than anticipated. This caused delays in other parts of the study preparation, resulting in constraints and logistical challenges during recruitment and baseline testing. These issues were resolved in later visits and testing (V1, V2).

### Evaluation of tools for measurement

#### Blood tests and questionnaires

At baseline screening, the laboratory results were delayed for some participants causing a delayed inclusion for some participants, this was resolved at V1 and V2. Otherwise, both sampling and submission of the results were satisfactory.

#### Flexibility testing

Two physiotherapists performed comprehensive and time-consuming flexibility tests. The procedure and logistics worked well for both personnel and participants. In addition, the tests revealed that some participants needed time to change position, resulting in uncertainty about their functional capability for aerobic hall exercise sessions.

#### Endurance testing

Thirteen participants (81.3%) performed both endurance tests at BS and V2. Out of these 13 subjects, 8 (BS) and 7 (V2) fulfilled the criteria for reaching V̇O_2max_.

The logistics and test procedures worked fairly well for both personnel and participants. One participant had problems with entering the treadmill, and the test personnel therefore made a small staircase to facilitate the entrance. However, the examiners experienced that the time scheduled for each participant was too short, suggesting each test be extended by 5–10 min.

#### Strength testing

A total of three examiners were involved. One examiner performed the strength testing at all three stations for the same participants. The logistics worked well for both the examiners and participants. The test procedures need improvement to simplify interpretation of test results and to simplify transfer to data file storage.

#### Use of activity tracker

All 16 participants wore the AT during the 6-month exercise intervention period for an average 127 days (95% CI 69.0, 185.0). Fourteen participants continued to share PA data from the AT for six additional months with an average 160 valid days (95% CI 26.7, 293.3). Mean number of valid days from the full year of recording, when including all participants, was 289 (95% CI 116.5, 461.5). Five (30%) participants did not own a smartphone. The ATs were connected to participant’s own smartphones or to a project smartphone for data synchronization and downloading. Although several participants sporadically forgot to synchronize data, no data were lost due to high storage capacity in the AT.

### Feasibility criteria achievement

Table 8 summarizes achievement of the pre-specified recruitment and enrolment criteria. In general recruitment performed fairly well with need for some improvement.

**Table 8:** Recruitment criteria achievement

Evaluation criteria	Goal	Result (95% CI)
Recruitment process (*n*=75)		
Response to postal invitations	> 0.25	0.27 (0.18, 0.38)^a^
Eligible after telephone screening	> 0.20	0.21 (0.14, 0.32)^a^
Eligible after baseline screening	> 0.18	0.21 (0.14, 0.32)^a^

^a^95% CI indicates suboptimal performance

Table 9 shows pre-specified adherence and side-effects feasibility criteria achievement. We experienced that participation was somewhat lower during the winter due to seasonal flu. Retention was excellent and there were no side-effects.

**Table 9:** Adherence and side-effects criteria achievement

Evaluation criteria	Goal	Result (95% CI)
Adherence and side-effects (16 participants, 41 sessions)		
Exercise sessions mean attendance	> 0.75	0.70 (0.60, 0.80)^b^
Dietary counselling session mean attendance	> 0.80	0.75 (0.51, 0.90) ^b^
Habit change counselling sessions mean attendance	> 0.80	0.79 (0.55, 0.92) ^b^
Attrition from study	< 0.20	0^a^
Dropouts due to exercise-induced injury	< 0.10	0^a^

^a^Performed well

^b^Needs improvement

### Interviews

The interviews showed that the participants were highly motivated for lifestyle changes when they joined the intervention. Weight loss wishes and the feeling of being in poor shape were frequently mentioned as reasons for this. The participants expressed overall satisfaction with the study plan and especially the exercise sessions. However, some participants suggested alterations, such as more dietary counselling later in the intervention period, in order to raise their consciousness about healthy diet.

The social part of the intervention was highlighted as particularly important for attending the exercise sessions. The participants described how support from the instructors, as well as their peers, encouraged them to attend the sessions and make their best effort when they exercised and expressed a strong feeling of being in this together.

According to interviews that were performed at V3, a few participants managed to maintain some of the lifestyle changes they had made during the study. Many participants found it hard to maintain high levels of physical activity when the intervention ceased. Frequently mentioned reasons for not keeping up the activity were injuries and pain from musculoskeletal conditions. Some said that lack of time and a busy schedule at work prevented them from maintaining new habits or staying active. Several participants described that being “on their own” was a significant barrier for being active and expressed that being in a group made it far easier to become and stay active.

Findings from the qualitative interviews will be further elaborated in a separate paper.

### Unintended consequences

#### Balance testing

Due to a communication failure within the study team, the balance test was not incorporated in the test scheme and therefore not performed.

#### Test of rate of force development» (RFD)

The measurement of RFD was done 6 weeks after BS due to a technical problem at the test site. The RFD test at V2 was done according to protocol. After testing, we revealed a test protocol error implying that the test load at V2 was based on 1RM from strength test at V2, when the correct load should have been from the first test 6 weeks after baseline. The results were therefore difficult to interpret, and the protocol will be corrected for the planned RCT.

#### Group exercise protocol adherence

We observed that participants, even when receiving visual aid and instructor supervision, experienced difficulties in following prescribed exercise programs, particularly at higher intensity levels. Deviations from protocol were significantly attenuated towards the end of the study period, for time in the light HR zone, but not for higher intensities. Data from this is presented in an abstract elsewhere [59] and will be further analyzed in a future paper.

#### Participants’ daily activity encouragement

We planned that instructors at the end of every group session would encourage participants to increase their daily activity. However, instructors experienced that the participants were overwhelmed by the project activity and not responsive for more input during the first months. This was not mediated to the project coordinator. In the RCT we plan for a more structured communication between the project coordinator and instructors and also more structured feedback to the participants to encourage to more daily activity. We also plan to implement an app that regularly release short “nudging” messages on the participants’ smart phones.

## Discussion

This study aimed to evaluate feasibility and inform planning of a large RCT to facilitate and maintain a healthier lifestyle among high-risk older adults, the RESTART trial. Achievement of the pre-specified primary feasibility criteria (Table 7), and participant interviews during and after the intervention formed the basis for the evaluation. There were no attrition or dropouts from the study due to exercise-induced injury, indicating that the intervention is safe. The recruitment response criteria were met, but wide confidence intervals indicate that the recruitment strategy needs improvement. Activity trackers were well tolerated and gave valuable information. Attendance was somewhat lower than our pre-specified goal, and several measures will be taken to improve adherence in the future RCT. Participant responses during interviews supported the general study outline and provided important suggestions for improvement. We therefore conclude that a full-scale RCT is feasible but will need refinement and improvement regarding some recruitment and adherence aspects that are fully achievable.

### Strengths

The Tromsø Study is a large ongoing cohort study conducted in the municipality of Tromsø, Northern Norway. Response rates are generally high [60] and 65% of those invited to the Tromsø 7 survey participated, reflecting a positive attitude to research. However, to recruit older participants with obesity and low PA levels to participate in a clinical trial is challenging [61, 62]. The recruitment was satisfactory, indicating that the Tromsø Study is a good recruitment base for a larger RCT. This was also pointed out by participants during interviews.

People in the included age-group frequently have co-morbidities, which increases their vulnerability to adverse events following HIIT. This feasibility study indicates that group-based heavy strength training and HIIT is well tolerated and effective in this group, given flexibility in exercise planning and guidance of participants. This is in line with another feasibility study of older adults [63].

According to a systematic review by Falck et al. [64], a majority of studies of PA among older adults used self-report measures, with low validity and reliability. In this study, we objectively measured PA using an accelerometer device for 1 week at baseline and at end of intervention. In addition, participants were equipped with a Polar M430 AT, recording up to one year of daily PA measurements. Wearing the AT was feasible for most participants who registered data on most days. One participant experienced eczema from wearing the AT but continued to wear it by own choice.

Conducting a large RCT with many different and coordinated elements requires an experienced research infrastructure. The CTU at UNN has extensive trial experience. The study also included less experienced test sites and personnel, we therefore experienced logistic challenges during baseline testing, which we corrected for in the next test round.

### Lessons learned

#### Recruitment response

Timely recruitment of sufficient participants is an important success criterion [65], which has consequences for participants, researchers, funding bodies, and policy makers [66]. Recruitment of participants to clinical trials is challenging [67, 68], and recruitment of elderly into exercise trials is even more difficult [62]. To set a modest and realistic target for recruitment in an RCT, it is crucial to adhere to trial timeline. Even though our recruitment using postal invitations is comparable with other studies [61], we plan for a more comprehensive recruitment for the RESTART trial. This will include telephone reminders, use of promotion material for social media, and more extensive use of mass media.

Participants were recruited based on Tromsø 7 survey examination results in 2015–16. As time between the Tromsø 7 survey and recruitment for other studies increases, the health status of potential participants may change, and some will no longer meet the trial inclusion criteria. In the present study, two participants had lost considerably weight since the Tromsø 7 survey and were borderline to the inclusion criterion of BMI ≥ 30 kg/m^2^. During the processing of baseline questionnaires, it turned out that one participant had increased PA level significantly since the Tromsø 7 survey. These findings indicate that for a full-scale RCT, we will exclude those who have taken part in an exercise scheme in the past year. We also may need to extend our recruitment base to ensure enough participants are available for inclusion, since the time span from the Tromsø 7 survey has increased. This might indicate that we exclude elevated NORRISK 2 as an inclusion criterion, as BMI>30 kg/m^2^ and low activity level indicate a higher risk of future cardiovascular events.

The telephone screening was successful and captured the eligibility criteria quite well, but there is room for improvement. To assess individual functional capability more precisely however, we need to ask more specific questions in a future RCT, e.g., examine the ability to move quickly from sitting or lying position to standing position.

Baseline screening and functional assessment was informative for planning of the exercise sessions.

#### Endurance testing

Testing VO_2max_ as a measure of aerobic exercise capacity is demanding for this group of participants. Applying RER ≥ 1.05 as one of the criteria for reaching V̇O_2max_ during the test is debated. Edvardsen et al. found in a Norwegian multicentre study that RER-value indicating exhaustion varied in relation to both age and fitness level and defined RER-limits > 1.05 (under 65 years) or >1.00 in age 65+ [35]. This age-related difference in RER cut-off value may be explained by the typical difference in muscle fibre composition between young and older subjects, with older subjects commonly displaying a lower proportion of type II fibres. In turn, since fewer type II fibres likely will result in less lactic acid formation, and RER may be reduced. Yet, other authors argue for higher cut-offs [69]. During treadmill endurance testing, we found that more than half of the participants fulfilled the criteria for V̇O_2max_. This finding is in line with other studies [70]. Other factors than muscle fibre composition that could explain why only 50% fulfilled the V̇O_2max_ criteria is that older subjects may experience pain and/or have other health-related limitations for pushing themselves to exhaustion. A measure of change in V̇O_2max_ is still of high value, as it is documented to be strongly associated with cardiovascular health [71].

We consider including the “Time Up &Go-test” for baseline screening and follow-up in in the RESTART trial. This test would provide information about the TUG predictive value for future falls in older adults when standardized testing conditions and control of the significant potential confounders [72].

Any hearing-impairment must be detected, as the exercise intervention requires participants to hear and respond quickly to the instructor´s messages. The inclusion process provided important experiences about older participants with impaired functional capacity.

After power calculation for the full-scale RCT we plan for 55 participants in the intervention group, divided into 4–5 exercise sub-groups. Clinical trials are susceptible to self-selection bias, which hampers the generalizability of their results. The present study and the planned RESTART trial are particularly prone to self-selection bias, as study participation is demanding on an individual level. Nevertheless, the feasibility study and RESTART trial aims to develop and test a model for behavioural change among motivated older adults with high internal validity that can be generalized to comparable populations.

#### Study conduct

It is important to capture relevant questionnaire data from participants in a clinical trial. Most participants filled out a time-consuming comprehensive questionnaire at three time points during the feasibility study, showing that that the questionnaires were manageable. However, in the full-scale RCT we will strictly reconsider questions that can be eliminated.

The attendance to the group sessions was somewhat lower than the pre-specified feasibility criteria goal, but the result is still within the 95% CI. In this selected group of participants, one might consider a slightly lower mean attendance to be acceptable. We experienced that participation was somewhat lower during the winter due to seasonal flu.

The use of experimental data is important to understand factors that influence adherence. Exploring participants’ experiences by semi-structured interviews and focus group sessions revealed that the social group environment was important for adherence and to maintain motivation for follow-up of the study regimen. Interviews also contributed valuable insight into the participants’ experiences and attitudes towards lifestyle changes, and thus, a more comprehensive understanding of participants’ motivation and barriers for a lasting lifestyle change.

Aging often brings decreased social support, networking, and interaction, which further augments physical inactivity and detrimental nutrition patterns [73, 74].

In the planned RCT, we will build and encourage social network related to group activities and establish peer-support strengthened with smartphone application. Therefore, we will change location for the group activities to a training centre with a spacious rest hall facilitating social interaction and networking before and after the sessions.

#### Follow-up

Many lifestyle interventions fail to demonstrate maintenance of long-term effects, as participants with obesity and high CVD risk frequently return to baseline weight and PA levels after intervention end [75, 76]. One of four feasibility study participants maintained their lifestyle 6 months after the intervention ended. Following up on interview responses, we plan the RESTART trial with a longer intervention period, to strengthen and consolidate new habits and social bonds between participants and to downscale the intervention towards cessation.

#### Intervention elements

We expected that the feasibility study participants with obesity, low activity levels, and elevated CVD risk were susceptible to training overload and injuries. An important observation was that participants tolerated the HIIT exercise well, as there were no dropout due to injuries. A few participants needed short-term alternative exercises, and some consulted their own physician for advice. We experienced that general practitioners’ advice were highly variable. To secure coherent and evidence-based advice, we aim to have a project-associated medical doctor with high competence in sports medicine in the RESTART trial.

One systematic review has pointed out that outdoor PA in natural environments can be beneficial for mental wellbeing [77]. This might facilitate long-term maintenance of exercise habits, but there is a need for evidence from well-designed trials to expand our understanding of effects on mental and physical wellbeing. We planned outdoor activities during the last part of the exercise intervention in the feasibility study. Those who participated in HIIT uphill walking activity gave very positive feedback in the interviews, but we also learned that winter conditions are challenging for participants who lack outdoor equipment. In the RESTART trial, we plan for more outdoor activities. We will also consider including outdoor winter equipment to the participants as a bonus for participating.

Participant attendance was slightly lower than 80% to dietary and habit change group sessions. However, according to a unison interview response, participants needed the first 3 months to get familiar with, and follow up, the exercise group sessions. The participants further questioned the value of the nutritionist’s and psychologist’s advice, given their own prior experiences from previous lifestyle change attempts. Hence, in the RESTART trial, we will introduce the dietary and habit change counselling group sessions later in the intervention and in a stepwise approach to prevent overload intervention activity and input. To increase the learning outcome, we plan for one more individual dietary counselling session, and add one practical session with cooking, altogether four sessions.

Individuals with obesity may have their own personal story of failure and feeling of shame and must be met with respect and understanding. In the habit change counselling group sessions, we used self-regulation theory by Implementation Intentions (IIs) [51]. To enforce habit change in the RESTART trial, we plan five 2-h group sessions, combining IIs with Acceptance and Commitment Therapy (ACT) [78]. Both methods are transdiagnostic and can be implemented across a range of therapeutic settings. ACT utilizes concepts of mindfulness and acceptance to identify values and goals in life and is proven effective among older adults [79]. By this approach, we aim to increase the likelihood that participants actually establish their new health-facilitating behaviour in their everyday life as healthy habits.

#### Measurement of physical activity

A systematic review has pointed out that most studies of PA among older adults have used self-report measures, suffering from poor validity and reliability [64]. In the present study, we examined two different methods to measure PA objectively. A hip-worn accelerometer (ActiGraph wGT3X-BT) was used for 1 week at BS and 1 week at V2. We also collected daily PA data for the entire duration of the study and during 6 months of follow-up using a Polar M430 AT, with a mean average wear time of nearly 80%. This indicates that the AT was mostly well tolerated and provided valuable data regarding participants’ PA intensity levels and daily activity [80]. In a previous study, we have also shown that Polar M430 is valid to measure total energy expenditure [81].

Going forward, in the planned RESTART trial, we will place additional focus on guidance and training the participants in using HR monitors. As we observed varying degrees of adherence to the prescribed exercise HR zones, we will also analyze data from the exercise sessions continuously during the project period, to provide more frequent feedback and ensure that participants reach the desired intensity level during exercise.

## Conclusions

The present feasibility study forms the basis for the full-scale RESTART randomized trial. We experienced no dropouts from the feasibility study due to exercise-induced injury, indicating that the intervention is safe. Minor logistic improvements were implemented during the intervention. We plan for achievable improvements regarding participant recruitment and adherence and will adjust the schedule and content of the dietary and psychological counselling. We conclude that it is feasible and practical to conduct a full-scale RCT of a multi-component intervention among sedentary older people with obesity and elevated cardiovascular risk.

## Data Availability

Participants were few and there was a lot of information collected for each person. To limit the risk of reverse identification of de-identified sensitive participant information, data will not be made publicly available. Data may be obtained from the main author upon reasonable request.

## Abbreviations

ACT: Acceptance and Commitment Therapy
AT: Activity tracker
RM: One repetition maximum (maximal muscular endurance)
BMI: Body mass index
BS: Baseline screening
CVD: Cardiovascular disease
CTU: Clinical Trial Unit
ECG: Electrocardiogram
HIIT: High-intensity interval training
HR: Heart rate
IIs: Implementation Intentions
PA: Physical activity
RCT: Randomized controlled trial
RER: Respiratory exchange ratio
RFD: Rate of force development
